# Fast quantifying collision strength index of ethylene-vinyl acetate copolymer coverings on the fields based on near infrared hyperspectral imaging techniques

**DOI:** 10.1038/srep20843

**Published:** 2016-02-15

**Authors:** Y. M. Chen, P. Lin, Y. He, J. Q. He, J. Zhang, X. L. Li

**Affiliations:** 1 College of Electrical Engineering, Yancheng Institute of Technology, Yancheng, 224051, P.R. China; 2 College of Biosystems Engineering and Food Science, Zhejiang University, 866 Yuhangtang Road, Hangzhou 310058, P.R. China

## Abstract

A novel strategy based on the near infrared hyperspectral imaging techniques and chemometrics were explored for fast quantifying the collision strength index of ethylene-vinyl acetate copolymer (EVAC) coverings on the fields. The reflectance spectral data of EVAC coverings was obtained by using the near infrared hyperspectral meter. The collision analysis equipment was employed to measure the collision intensity of EVAC materials. The preprocessing algorithms were firstly performed before the calibration. The algorithms of random frog and successive projection (SP) were applied to extracting the fingerprint wavebands. A correlation model between the significant spectral curves which reflected the cross-linking attributions of the inner organic molecules and the degree of collision strength was set up by taking advantage of the support vector machine regression (SVMR) approach. The SP-SVMR model attained the residual predictive deviation of 3.074, the square of percentage of correlation coefficient of 93.48% and 93.05% and the root mean square error of 1.963 and 2.091 for the calibration and validation sets, respectively, which exhibited the best forecast performance. The results indicated that the approaches of integrating the near infrared hyperspectral imaging techniques with the chemometrics could be utilized to rapidly determine the degree of collision strength of EVAC.

The plastic-covered agricultural greenhouses can preserve the moisture of the atmosphere inside the greenhouse in the dry climate, increase the carbon dioxide concentration, effectively resist the outside cold air and threat of pest and weed to the crop, which can provide crops with a comfortable growth environment with relatively suitable humidity and temperature, as well as enable the extension of cultivation in terms of the growing season and the location through the use of protective greenhouse films^1^,^2^,^3^. It has been proved that the ripened fruit yields of some crops growing in the agricultural greenhouses coverings such as strawberries, peppers, cucumbers, tomatoes generally increased to more than 28%^4^,^5^. By the year 2009, only in China, the country with the greatest greenhouse area in the world, 1,000,000 ha were covered with greenhouse and tunnel plastic films. In addition, worldwide, the agricultural plastic film market alone was estimated to be worth $5.87 billion in 2012^6^. At present, some main organic ingredients including poly vinyl chloride (PVC)^7^, linear low-density poly ethylene (LLDPE)^8^, and ethylene-vinyl acetate copolymer(EVAC)^9^ have been used to manufacture the plastic greenhouses films. The EVAC films are now the most widely used poly grade in horticultural practice, due to its relatively better optical properties than the PVC’s and the LLDPE’s, which can make the crop leaves in the greenhouse fully realize the photosynthetic reaction and stably physical and chemical properties under the harsh environmental conditions such as solar radiation and frost erosion during their use when special additives are generally added to the formulation of the materials^10^. In addition, the breakdown EVAC films can be easily refabricated and continue to be reused, which can effectively reduce environmental health hazards and cause a relatively competitive low market price^11^,^12^. Therefore, the EVAC films are competitive with the other related products in many farmland applications and received widespread attention and application in the modern agricultural production^13^.

The quality of plastic films is varied and essentially determined by raw materials, processing equipment and technology the enterprises used. How to fast and accurately determine the quality parameters of a large number of plastic film products has become a challenging project. Especially among them, the collision index will directly determine its lifetime. The traditional destructive physical experiments for evaluating the collision strength of EVAC coverings was extraordinarily time-consuming and inconvenient^14^, so it is not suitable for the practical application on the farmland and the rapid measurement of a wide variety of products in the rural markets.

Spectroscopy techniques provide an alternative to evaluating the quality of EVAC films in a fast and precise pattern. Chernev *et al.* successfully applied Raman spectroscopy to non-destructive determination of EVAC cross-linking degree in photovoltaic (PV) modules^15^. Hirschl *et al.* presented two potential optical methods of ultraviolet/visible and Raman spectroscopy for measuring the crosslinking degree of EVA encapsulants in-line in the PV module manufacturing process spectroscopy^16^. To the best of our knowledge, the current international spectroscopic investigation mainly focused on the determination of crosslinking quality of EVAC materials based on the ultraviolet/visible and spectroscopic techniques, however, seldom literature referred to the measurement of the collision strength using the near infrared (NIR) hyperspectral techniques.

The NIR hyperspectral techniques is considered as a powerful extension of an analytical technique to study the different quality attributes in a sample, resulting in many successful applications in the quality control of many other organic products^17^. The NIR hyperspectral techniques take advantage of the interaction between electromagnetic radiation emitted from lights and physicochemical materials existed in the organics. Generally, the responses of the electromagnetic overtones and combinations of absorptions of molecular bonds (C-H, C-C, C-O and C=O) in chemical substances of plastic materials could be discovered in the NIR spectra^18^. In this study, we assumed that there was a relationship between these chemical bonds and the collision strength of the EVAC materials. Thereby, the following experiments and specific aims of this survey were to (1) develop a novel strategy combining the hyperspectral imaging techniques with the chemometrics for fast quantifying the collision strength index of EVAC coverings to assess its service life, (2) explore a new model of selecting the optimal wavebands for boosting the accuracy and robustness of detecting the collision strength, (3) contribute to the EVAC manufacturers to better master and control the quality of the products of EVAC films.

The paper is organized as follows: Section 2 describes the materials and devices. Section 3 presents theory and approaches of random frog (RF)^19^, successive projection (SP)^20^, support vector machine regression (SVMR)^21^ and performance assessment. Section 4.1 and 4.2 explains the relationship between the absorption attributes of NIR spectroscopy and the inner chemical composition of EVAC materials. Section 4.3 gives the results of collision experiment. Section 4.4 constructs the quantitative chemometric model. Section 4.5 compared the modeling results by using different preprocessing algorithms. Section 4.6 discusses issues of selection of the characteristic wavelengths to enhance the forecast precision. Section 4.7 provides the results of predicting collision strength using fingerprint spectral variables. The conclusions are drawn in the last Section 5.

## Materials and Devices

**Figure 1:**
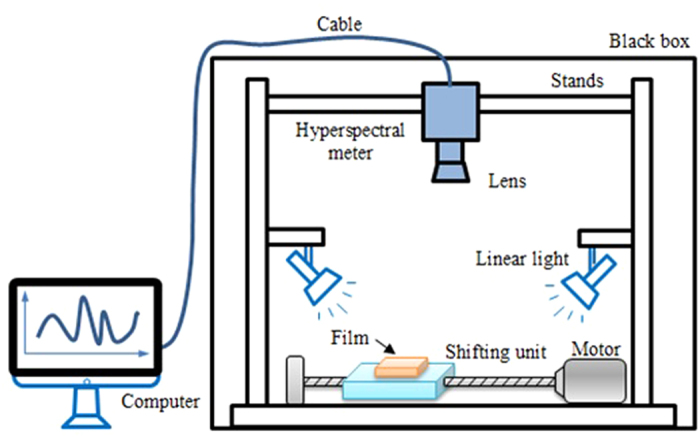
Block schematic diagram of the near infrared hyperspectral imaging system.

The products of EVAC films were provided by four different manufacturers from Weifang Shandong, Suzhou Jiangsu, Taizhou Zhejiang and Shunde Guangzhou, in China. The experimental samples were all fixed in the experimental fields in Dafeng, Yancheng. About one year later, these films were pulled down, taken back to the laboratory and measured by using the near infrared hyperspectral imaging meter and the collision strength analysis device. The main components of hyperspectral imaging systems consisted of: Fiber-Lite DC950 linear radiant illuminant (JennerIndustries Inc., USA), N17E-QE hyperspectral imaging meter (Spectral Imaging Ltd. Oulu, Spectral Imaging Ltd. Oulu, Finland), C-mount OLES22 extremely accurate lens (Specim, Spectral Imaging Ltd., Oulu, Finland), IRCP0076 motor-driven shifting platform (Isuzu Optics Corp, Taiwan, China). These devices were installed in a closed dark box and linked with the outside computer machine through a communication cable. The vertical height of the objective lens was tuned to 293 mm. The exposure time was adjusted to 2550μs. The mean velocity of shifting platform was set as 33 mm/s. The resolution of near infrared hyperspectral image was resized to 310 × 260 pixels. The resolution of wavelengths was 4 nm. The block schematic diagram of hyperspectral imaging systems was illustrated in Fig. 1.

The hyperspectral charge coupled device were used to gather the reflectance signal intensity, which coupled both the information of chemical composition of measured instances and the intensity of the linear light sources. In order to uncouple these two kinds of information, the reference intensity of black and white boards were used as the baseline for such separation purposes. The calibration information 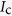 could be evaluated by using the following formula:


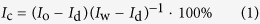


where, 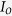 was the intensity values of raw hyperspectral image, 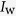
and 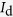 were the intensity values of the white and dark boards, respectively. The procedures of visualizing and calibrating hyperspectral data were implemented with the ENVI 4.6 (Exelis Visual Information Solutions Inc., USA), The raw coverings were cut into the square samples by the chop-out die before implementing the impact experiment. The impact experiment was carried out in terms of the items of assessing the performance of plastic angle collision strength of the People’s Republic of China light industry standard (QB/T 1130—91)^22^. The tearing degree of EVAC coverings was measured by using the collision strength analysis meter (XJ-300 A, Wuzhongshi Ltd., China). The main specifications were set as following: impact energy of 7.35 J, impact velocity of 3.8 m/s, pre-blowing angle of 150°, impact blade fillet of R2 ± 0.5 mm, Jaw radius of R-1 mm, pendulum shaft axis to the sample center distance of 395 mm and power of 380 V/1500 W.

## Theory and approaches

In this paper, two distinct kinds of spectral variable selection algorithms of RF^19^ and SP^20^ were conducted to extract the most important spectral wave bands to determine the collision strength index of EVAC. The RF algorithm is a dynamic process of simple random walk, which is considered as similar as the algorithm of reversible jump Markov chain Monte Carlo is implemented in an iterative way. The SP algorithm is explored to eliminate the collinear problems between the variables based on choosing variables with the minimal redundant information utilizing a simple projection operation in a vector space. The chosen characteristic variables of high-dimensional data will be used for the input space of the following learning machines. The learning algorithm of SVMR^21^ is a statistical learning theory who transforms the original input space into the higher-dimensional Hibert space based on the kernel functions, then implements the linear regression in such feature space. A correlation model between the significant spectral curves which reflected the cross-linking attributions of the inner organic molecules and the degree of collision strength was set up by taking advantage of the SVMR approach. Three parameters of the root mean square error (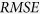), the square of percentage of correlation coefficient (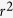) and the residual predictive deviation (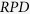) are applied to the assessment of model performance. In general, a good model with the values of low 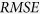 and high 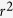 and 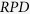 indicates a good prediction ability^23^. All the algorithms were implemented by using the Unscrambler X10 (CAMO Corporation, USA) and the Matlab R2014a (The Math Works, Natick, USA).

## Results and Discussion

### Chemical structures of EVAC

**Figure 2:**
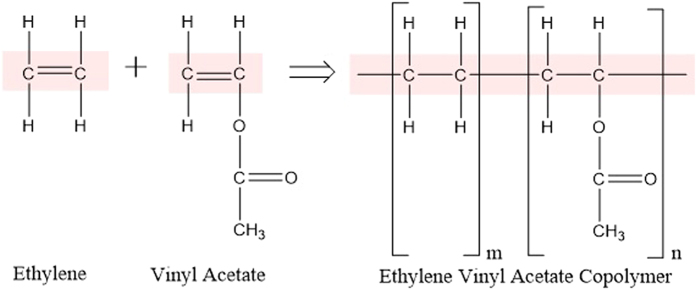
Chemical structures of ethylene and vinyl acetate monomers and poly (ethylene vinyl acetate).

Figure 2 showed the chemical structures of the EVAC ((C_2_H_4_)_m_(C_4_H_6_O_2_)_n_). It could be seen that the EVAC substance contained the functional groups of ethylene (-CH_2_- CH_2_-), vinyl (-CH_2_-CH-) and acetate (CH_3_-CO_2_-) and the bonds of C-H, C-C, C-O and C=O. The chemical groups and bonds of the EVAC directly determined the collision strength index of EVAC films^24^.

### Spectral features of EVAC

**Figure 3:**
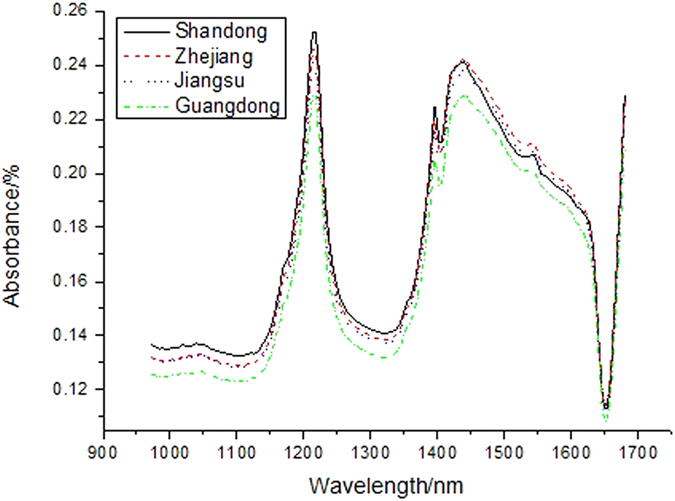
The characteristic absorbance spectra of the EVAC coverings in the NIR wavelength region of 972–1670 nm respectively manufactured in four different zones of Shangdong, Zhejiang, Jiangsu and Guandong provinces, in P.R. China.

The hyperspectral images were collected and calibrated by using Eq. 1. The spectra information was able to be extracted from each pixel positions of gathered multidimensional hyperspectral images of EVAC films. The scope of interests (SOI) function provided by the ENVI v4.6 software was implemented to choose the pixels of the EVAC hyperspectral image instances. The pixels outside such SOI would not be considered for further modeling application. The spectra of all pixels in such SOI were taken the average. A mean spectrum was then obtained and used to represent the corresponding measured sample. Four characteristic absorbance spectral curves of the EVAC coverings in the NIR wavelength region of 972–1670 nm respectively manufactured in four different zones of Shangdong, Zhejiang, Jiangsu and Guandong provinces, in P.R. China were illustrated in Fig. 3. The mean spectral curves extracted from hyperspectral images of the EVAC instances between the waveband range of 972–1670 nm were chosen for discussion in our following investigation.

The absorption peaks of the spectral bands were partial overridden, which caused that the average NIR spectral curves of EVAC materials in parts of spectral region were comparatively flat with some broadband peaks. The primary absorption bands existing in the NIR spectral scope were the strong overtone and combination absorptions of carbon, hydrogen and oxygen containing bonds (C-H, C-C, C-O and C=O)^25^, which presented at a number of characteristic wavelengths. For EVAC films, four local absorption peaks appearing at 1080, 1230, 1410, 1445, 1550 and 1680 nm were observed mainly due to the presence of the functional groups of ethylene, vinyl and acetate in the instance (see Fig. 3). The detailed assignments of NIR spectroscopic bands of EVAC were listed in Table 1.

### Physicochemical properties of EVAC

One important application of EVAC materials was used as the coverings of crops in the farmland in order to defense against the change of outside severe environmental factors^26^. The structures of molecules of greenhouse coverings were comparatively compact at the beginning of application. The oxygen or other molecules had difficulty to penetrate into the interior films. The experiment indicated that the collision strength rate of EVAC films provided by four different manufacturers all reached 100%. However, the EVAC sheds were covered on the ground in the open fields and straightforward corroded by multiple outside factors such as the damp-heat, UV-irradiation and corrosive atmosphere. The molecules of EVAC materials became flexible for a period of practical utilization. Several active molecules such oxygen would rapidly combine with the free radicals of films and break the molecule chains of EVAC^27^. Unfortunately, few investigations presented information on the changes in the interior structure of the EVAC films as a consequence of environmental exposure in the fields. The degradation processes took place at a molecular level. Thereby, the collision strength rate of films decreased. The summary of degrees of collision strength of four different kinds of greenhouse films being used for one year in our study was listed in Table 2.

There are a total 360 pieces of EVAC films for the further analysis, where each kind of products has 90 instances. For learning the relationship between the hyperspectral feature variables and the corresponding collision strength attributes, there were 70 and 20 films utilized for both calibration and validation purposes for each group, respectively.

### Construction of chemometric model

The absorption attributes of NIR spectroscopy can reflect the inner chemical composition of materials, in addition, the chemical molecules, to a large extent, determine the physical properties of materials^15^,^27^. This paper supposed that the change of the chemical groups and bonds would affect the intensity change of NIR absorption spectroscopy, so the intensity of absorption peaks of spectral curve could be used to estimate the collision strength index of EVAC films. In this survey, we attempted to take advantage of the techniques of NIR hyperspectral image techniques for rapidly forecasting the collision strength degree of EVAC materials. The chemometrics models were finally set up to evaluate the collision strength indices in terms of their corresponding spectral information. Each acquired mean spectral curve was employed to generate the predictors (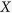), where each row of the matrix represented an independent observation and each column of the matrix represented wavelengths containing 211 individual spectral variables. In order to establish a meaningful regression model, it must be ensured that the row instances of responses (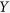) containing the properties of collision strength index corresponded to the row instances in 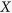. The multivariate calibration model was set up to correlate the spectral matrices and the collision strength indexes of distinct EVAC films. For the future unknown EVAC films, the established multivariate model could be used to estimate the collision strength properties straightly from the gathered spectra of pixels of EVAC films.

### Spectral data pre-processing

**Figure 4:**
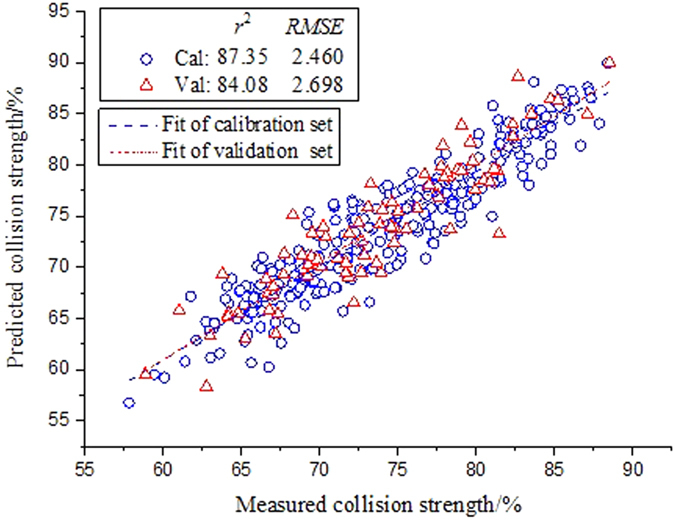
Measured versus predicted values of collision strength for the SG-1^st^-Deriv-based methods under the entire wavelength spectra.

The collected spectra of EVAC samples were vulnerable to the effects of the random noises, surface scattering light, stray light, etc. during the collected process^28^. Generally, the procedure of spectral data pre-processing was an indispensible step carried out before establishment of calibration model in order to eliminate such influence. Four different pre-processing algorithms including Savitzky–Golay smooth (SG), standard normal variate (SNV), SG first-order derivative (SG-1^st^-Deriv) and multiplicative scatter correction (MSC) algorithms were performed in order to enhance the modeling precision^29^. The performance of these pre-processing algorithms were compared by the subsequent built support vector machine regression (SVMR) model using the pre-processed spectra data in the whole spectral range. The forecast capability of the calibration model was assessed by the values of root mean square error of cross validation (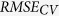) and prediction (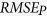), the correlation coefficients of cross validation (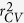) and prediction (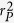) and 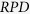. As shown in Table 3, the SG-1^st^-Deriv based method obtained the best prediction performance of 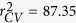, 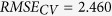, 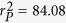, 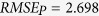 and 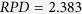 compared with the others. The results of predicting the collision strength based on the SG-1^st^-Deriv approach was illustrated in Fig. 4, where the symbols of circle and triangle denoted the calibration and validation set, respectively. Nevertheless, not all the preprocessing methods could boost the accuracy of prediction model. It could be seen in Table 2 that the 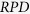, 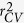, 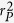, 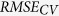 and 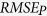 attained by the SNV based algorithm decreased by 5.10%, 1.62%, and 0.78%, and increased by 5.86% and 4.32% compared to the unpreprocessed, respectively. Thereby, all the subsequent forecast models were explored based on the SG-1^st^-Deriv pre-processed spectral data.

### Extracting fingerprint wavelengths

Hyperspectral imaging device usually was assembled high wavelength resolution sensors, which could record a high dimension of spectral information over a continuous range^30^. The multivariate statistics approaches could be directly utilized to deal with the big data sets of spectra. However, the raw spectra often consisted of a great deal of useless and redundant information, which would influence the predictive precision of calibration model. In addition, the large-scale dimension of spectral data also caused a slow modeling process. Hence, it was suggested to screen out the useful characteristic variables for the further modeling process. Furthermore, by this means, the selected feature variables could be used as a wizard to account for both the hyperspectra and response. Besides, the fingerprint wavebands could be employed to exploit cheaper multispectral imaging devices for the objective of practical farmland application. Thus, the procedure of uninformative spectral variable elimination was demanded to be carried out in order to accelerate the modeling efficiency and promote the forecast precision and stabilization^31^

**Figure 5:**
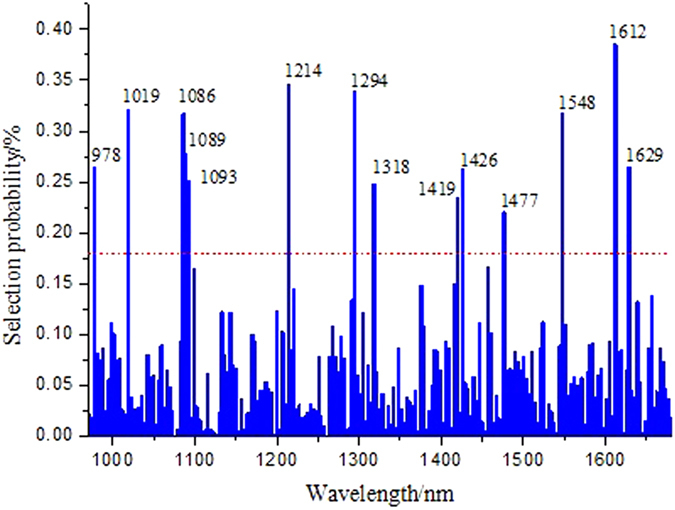
Selection probability of each wavelength variables by the algorithm of random frog.

In this survey, the fingerprint wavelengths representing the spectral feature for forecasting the degree of collision strength were evaluated by using the variable selection algorithms of RF and SP. Both algorithms for variable selection were carried out based on the calibration set. The parameter of 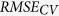 was employed to determine the optimal number of selected variables. For RF algorithm, there were two important parameters where one was the number of generations 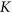, and the other was the initialized variables 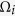 needed to be configured before implementation (see Section 3.1). Generally, the larger the generations 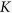 were, the more likely the RF algorithm was to find out the optimal variable subset. However, the massive generations would probably lead to an overfitting and a great operation expense^32^. Compromising both performance and operation expense, the generations 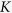 was set to 8000 in this survey. In addition, for the number of initialized variables 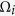, it was found that the values affected the iterative behavior merely during the initial process, but did not significantly affect the final algorithm performance^32^. The threshold of selection probability was fixed to 0.18 marked by the red dash line in Fig. 5 based on the experimental results. The wavelength variables whose values of selection probability were larger than such threshold were chosen as the fingerprint wavelength variables for subsequent calculation. There are fourteen wavelength variables (978, 1019, 1086, 1089, 1093, 1214, 1294, 1318, 1419, 1426, 1477, 1548, 1612 and 1629 nm) chosen by the RF algorithm. The spectral reflectance around 978 nm was caused by the O-C=O deformation of acetate. The spectral reflectance around 1019 nm was generated by the C-C streching of > HC-CH_2_ of vinyl. The spectral reflectance around 1086 and 1093 nm was dominated by the asymmetric C-C stretching of amorphous (trans and gauche). The spectral reflectance around 1214 and 1249 nm was governed by the CH_2_ twisting of amorphous. The spectral reflectance around 1318 nm was produced by the CH_2_ wagging of amorphous. The spectral reflectance around 1419 nm was formed by the CH_2_ bending of crystalline. The spectral reflectance around 1426 nm was affected by the CH_3_ asymmetric bending of acetate. The spectral reflectance around 1477, 1548, 1612 and 1629 nm was regulated by the 2 × CH_2_ rocking of all-trans-(CH_2_)-_n_^25^. Those wavelengths would be applied to the fingerprint wavelengths to take the place of the whole wavelength spectra for evaluating the degree of collision strength. The number of effective wavelengths decreased from 211–14. A simplified calibration spectral model with a pretty smaller dimension of spectral variables was generated.

In addition, the SP algorithm was considered that it can reduce the wavelengths by avoiding repetition of information or redundancies^30^. Thereby, it was conducted for choosing the optimal candidate wavelength variables for representing the spectral characteristics. As a result, eleven wavelengths were screened out, which were 1009, 1022, 1146, 1183, 1217, 1372, 1399, 1416, 1554, 1571 and 1602 nm. The spectral reflectance around 1009 and 1022 nm was generated by the C-C streching of >HC-CH_2_ of vinyl. The spectral reflectance around 1146 and 1183 nm was forced by the CH_2_ rocking of crystalline. The spectral reflectance around 1217 was governed by the CH_2_ twisting of amorphous. The spectral reflectance around 1399 and 1412 nm was formed by the CH_2_ bending of crystalline. The spectral reflectance around 1554 nm was regulated by the 2 × CH_2_ rocking of all-trans-(CH_2_)-_n_. The spectral reflectance around 1602 nm was regulated by the C=O stretching of acetate. The chosen wavelengths by SP were then used as the input variables instead of the entire wavelength spectra consisting of 211 wavelengths to set up a new forecast model to determine the degree of collision strength. When the fingerprint wavebands were determined by the algorithm RF and SP, the volume of hyperspectral images was then condensed by employing those feature images corresponding to the appointed effective wavelengths. The optimized prediction models would be established by avoiding the curse of wavelength dimensionality^25^.

### Predicting collision strength using fingerprint spectral variables

**Figure 6:**
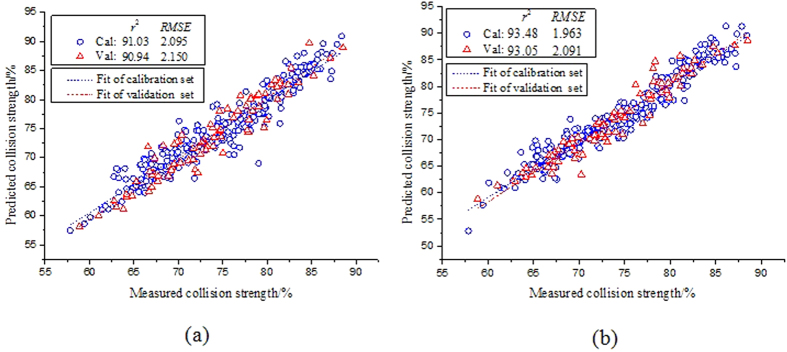
Measured versus predicted values of collision strength for both of (a) RF-SVMR and (b) SP-SVMR models under the extracted fingerprint spectra.

The algorithms of RF and SP were general strategy for the selection of wavelength variables. So, for a method to build a prediction model, this needed to be specified in order to implement them. The algorithm of SVMR was selected to set up the forecast model to the compressed spectral dataset. The reasons behind the choice was that it generally achieved significantly higher forecast accuracy compared with the traditional schemes in dealing with complex spectral data. The SVMR is a nonlinear calibration algorithm, which takes advantage of kernel trick to map the data input space to a high-dimensional feature space which was used to establish the quantitative relationship between the predictor matrix(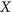) of the feature spectral dataset with the whole waveband scope (211 independent spectral reflectance variables) and the response vector (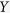) of the reference attribute value of collision strength. The Gaussian radial basis function (RBF) of 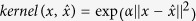 was the recommended kernel function compared with other kernel functions such as hyperbolic tangent and homogeneous polynomial kernels for the regression analysis in most of previous scientific literature^33^, probably because its compact support property was apt to eliminate the impact of nonlinear structures of dataset to a great extent in the infinite dimensional Hilbert space with less the generalization error^34^. When the RBF was chosen as the kernel function of SVMR, the final prediction performance of model, to a great extent, was determined by two important parameters of the regularization parameter of 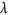 and the RBF kernel width of 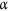. The grid-search technique was performed to optimize these two variables. The combination of parameter selection was verified by utilizing the approach of 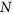-fold cross validation based on the instances in the calibration dataset. In this investigation, the highest cross-validation forecast precision was obtained, when the pair of parameters of 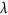 and 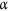 were set as 67.31 and 0.037, respectively. The experimental results of forecast precision were listed in Table 4. The rest 80 instances were used to comprise the forecast set only with the corresponding designated fingerprint wavebands. It could be seen in Table 4 that the forecast results gained by the SP-SVMR model was preferable to the individual SVMR and RF-SVMR models. The percentage of 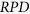, 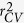, 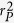, 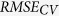 and 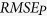 calculated by the SP-SVMR method increased by 28.99% and 2.81%, 7.02% and 2.69%, 10.67% and 2.32%, and decreased by 20.20% and 6.30%, 22.50% and 2.74% compared to the methods of individual SVMR and RF-SVMR, respectively. It could be concluded that the fingerprint wavelengths comprised the most important information concerning the forecast, which led to being more efficient than whole spectral wavebands. The predicted results of collision strength for both of RF-SVMR and SP-SVMR models under the extracted fingerprint spectra were illustrated in Fig. 6. The experimental results based on the methods of fingerprint spectral wavelength selection might contribute for developing the more accurate, economic and practical multispectral imaging equipment comprised a limited numbers of designated optical filters. Such technology could be further used to monitor the manufacturing process of EVAC films and assess the quality of the products for the target of farmland application.

## Conclusions

Because the traditional physical approaches for evaluating the collision strength index of EVAC coverings was extraordinarily time-consuming and inconvenient, the potential of combination of the near infrared hyperspectral imaging techniques with chemometrics for fast and convenient detection of such index was explored. The proposed methods could be used to predict the collision strength degree of EVAC materials quantitatively due to its ability to provide spectral information related to chemical components. The outcomes attained validation parameters of 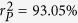, 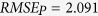 and 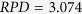. It was indicated that the hyperspectral imaging technology could be utilized to reveal the relationship of the collision strength attributes of EVAC materials and intrinsic structural changes of chemical groups in the copolymers. The final prediction performances were able to be accepted. This technique would be more suitable for practical application than the other physical or chemical measurements, in virtue of their advantages of more rapid, low cost, and non-invasive measurement, and convenient operation. Besides, the model (SP-SVMR) of selecting feature wavebands was verified to be quite significant for boosting the accuracy and robustness of the forecast models for detecting the collision strength. Due to more than 94 percentage of irrespective wavelengths removed, the percentage of 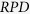, 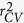, 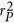, 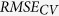 and 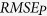 calculated by the SP-SVMR method increased by 28.99% and 7.02%, and 10.67%, and decreased by 20.20% and 22.50% compared with the model of using the full wavebands, respectively. The successful results of this experiment would contribute to the EVAC manufacturers to better master and control the quality of the products of EVAC coverings, which would bring about incremental competitiveness in international and domestic markets. In addition, the rapid determination of collision strength degree of EVAC coverings would ensure the labelling of such index accurately on the specifications of retail EVAC products.

## Additional Information

**How to cite this article**: Chen, Y. M. *et al.* Fast quantifying collision strength index of ethylene-vinyl acetate copolymer coverings on the fields based on near infrared hyperspectral imaging techniques. *Sci. Rep.* **6**, 20843; doi: 10.1038/srep20843 (2016).

## Figures and Tables

**Table 1:** Assignments of NIR spectroscopic bands of EVAC.

Assignments	Bands	Features
1000	O-C=O deformation	due to acetate
1016	C-C streching of >HC-CH_2_	due to vinyl
1060	asymmetric C-C stretching	due to all-trans-(CH_2_)-_n_
1080	asymmetric C-C stretching	amorphous(trans and gauche)
1170	CH_2_ rocking	crystalline
1220	CH_2_ twisting	amorphous
1330	CH_2_ wagging	amorphous
1410	CH_2_ bending	crystalline
1430	CH_3_ asymmetric bending	due to acetate
1435	CH_2_ bending	anisotropic
1450	2 × CH_2_ rocking	due to all-trans-(CH_2_)-_n_
1550	2 × CH_2_ rocking	due to all-trans-(CH_2_)-_n_
1680	C=O stretching	due to acetate

**Table 2:** The summary of degrees of collision strength of four different kinds of greenhouse films being used for near one year.

Manufacturers	Collision strength degree(%)			
	Mean	Standard deviation	Maximum	Minimum
Shangdong	67.14	3.82	77.72	57.82
Zhejiang	70.33	3.58	79.49	60.09
Jiangsu	76.43	3.81	87.22	64.79
Guangdong	82.01	3.17	88.50	74.57

**Table 3:** Forecasting the degree of collision strength from the raw and preprocessed spectroscopic data using the SVMR model.

Methods	Calibration set		Prediction set		
	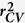	*RMSE*_*CV*_	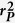	*RMSE*_*P*_	*RPD*
Raw	83.76	2.713	79.23	2.915	1.824
SG	84.39	2.526	80.27	2.823	1.945
SNV	82.40	2.872	78.61	3.041	1.731
SG-1^st^-Deriv	87.35	2.460	84.08	2.698	2.383
MSC	85.15	2.493	81.68	2.754	2.152

**Table 4:** The results of using the SVMR and RF and SP wavelength selection algorithms for determining the degree of collision strength in both calibration and prediction processes.

Models	Calibration set		Prediction set		
	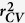	*RMSE*_*CV*_	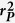	*RMSE*_*P*_	*RPD*
SVMR	87.35	2.460	84.08	2.698	2.383
RF-SVMR	91.03	2.095	90.94	2.150	2.990
SP-SVMR	93.48	1.963	93.05	2.091	3.074
